# Excitation Functions of Tsallis-Like Parameters in High-Energy Nucleus–Nucleus Collisions

**DOI:** 10.3390/e23040478

**Published:** 2021-04-18

**Authors:** Li-Li Li, Fu-Hu Liu, Khusniddin K. Olimov

**Affiliations:** 1 State Key Laboratory of Quantum Optics and Quantum Optics Devices, Institute of Theoretical Physics, Shanxi University, Taiyuan 030006, China; 20181602001@email.sxu.edu.cn; 2 Collaborative Innovation Center of Extreme Optics, Shanxi University, Taiyuan 030006, China; 3 Laboratory of High Energy Physics, Physical-Technical Institute of SPA “Physics-Sun” of Uzbek Academy of Sciences, Chingiz Aytmatov str. 2^b^, Tashkent 100084, Uzbekistan

**Keywords:** excitation functions of related parameters, participant parton, kinetic freeze-out temperature, transverse flow velocity, 12.40.Ee, 13.85.Hd, 24.10.Pa

## Abstract

The transverse momentum spectra of charged pions, kaons, and protons produced at mid-rapidity in central nucleus–nucleus (AA) collisions at high energies are analyzed by considering particles to be created from two participant partons, which are assumed to be contributors from the collision system. Each participant (contributor) parton is assumed to contribute to the transverse momentum by a Tsallis-like function. The contributions of the two participant partons are regarded as the two components of transverse momentum of the identified particle. The experimental data measured in high-energy AA collisions by international collaborations are studied. The excitation functions of kinetic freeze-out temperature and transverse flow velocity are extracted. The two parameters increase quickly from ≈3 to ≈10 GeV (exactly from 2.7 to 7.7 GeV) and then slowly at above 10 GeV with the increase of collision energy. In particular, there is a plateau from near 10 GeV to 200 GeV in the excitation function of kinetic freeze-out temperature.

## 1. Introduction

High-energy collider experiments are designed to study the strongly interacting matter at high temperatures and densities [1]. The deconfinement of colliding hadrons into quark-gluon plasma (QGP), which then rapidly expands and cools down [2], is conjectured to be created at such extreme collision energies [3,4,5,6]. In high-energy and nuclear physics, the study of transverse (momentum ($p_{T}$) or mass ($m_{T}$)) spectra of charged particles produced in nucleus–nucleus (AA) collisions is very important. In particular, the AA collision process at the Relativistic Heavy Ion Collider (RHIC) and the Large Hadron Collider (LHC) provides a good opportunity to study the signals and characteristics of QGP generation, so as to indirectly study the system evolution and the reaction mechanism of particle generation.

During the time evolution of collision system [7,8,9], the stages of kinetic freeze-out and chemical freeze-out are two important processes. In the stage of chemical freeze-out, a phase transition from QGP to hadrons occurred in the system, so the composition and ratio of various particles remain unchanged. In the stage of kinetic freeze-out, elastic collisions among particles stop, so their $p_{T}$ and then $m_{T}$ spectra are unchanged [8,10]. Therefore, by studying the $p_{T}$ ($m_{T}$) spectra, we can obtain some useful information, such as the effective temperature (*T*), the chemical freeze-out temperature ($T_{ch}$), and the kinetic freeze-out temperature ($T_{0}$ or $T_{kin}$) of the system, as well as the transverse flow velocity ($\beta_{T}$) of the final state particles. The temperature in which we do not exclude the contribution of transverse flow is called the effective temperature, which is related to the kinetic freeze-out temperature. The temperatures in the stages of chemical and kinetic freeze-outs are called the chemical and kinetic freeze-out temperatures, respectively.

It is very important to study the behavior of $T_{0}$ and $\beta_{T}$ due to their relation to map the phase diagram of Quantum Chromodynamics (QCD), though $T_{ch}$ is usually used [11,12,13,14,15,16] in the phase diagram. In order to extract $T_{0}$ and $\beta_{T}$, and study their dependence on energy, we can analyze the $p_{T}$ ($m_{T}$) spectra of particles using different models. These models include, but are not limited to, the blast-wave model with Boltzmann–Gibbs statistics [17,18] or Tsallis statistics [19,20,21], as well as other alternative methods [22,23,24,25,26] based on the standard distribution or Tsallis distribution. Here, the standard distribution denotes together the Boltzmann, Fermi–Dirac, and Bose–Einstein distributions. The alternative method regards the intercept of *T* versus $m_{0}$ as $T_{0}$, and the slope of $\langle p_{T}\rangle$ versus $\bar{m}$ as $\beta_{T}$, where $m_{0}$, $\langle p_{T}\rangle$, and $\bar{m}$ denote the rest mass, mean $p_{T}$, and mean energy of the given particles, respectively.

In our recent work [27,28], the blast-wave model with Boltzmann–Gibbs statistics or Tsallis statistics and the standard distribution have been used to analyze the spectra of particles produced in high-energy proton–proton (pp) and AA collisions. The related parameters were extracted and their excitation functions were obtained. Not only the blast-wave model [17,18,19,20,21], but also the alternative method [22,23,24,25,26], can be used to extract $T_{0}$ and $\beta_{T}$, though an effective temperature *T* is used in the latter. The alternative method is partly a new one, in which the extractions of both $T_{0}$ and $\beta_{T}$ are based on *T* [22,23,29] and the related derived quantities such as $\langle p_{T}\rangle$ and $\bar{m}$.

Due to the importance of $T_{0}$ and $\beta_{T}$ and their excitation functions, we use a new method in the framework of multisource thermal model [30] to describe the $p_{T}$ ($m_{T}$) spectra of identified particles in this work. Considering the contributions of two participant (contributor) partons to $p_{T}$ of a given particle, we regard the two contributions as the two components of $p_{T}$. The $p_{T}$ ($m_{T}$) spectra of identified particles (concretely charged pions, kaons, and protons) produced at mid-rapidity (mid-*y*) in central AA collisions which include gold–gold (Au-Au) collisions at the Alternating Gradient Synchrotron (AGS), lead–lead (Pb-Pb) collisions at the Super Proton Synchrotron (SPS), Au–Au collisions at the RHIC, and Pb–Pb and xenon–xenon (Xe-Xe) collisions at the LHC are studied. The center-of-mass energy per nucleon pair, $\sqrt{s_{NN}}$, considered by us is from 2.7 GeV to 5.44 TeV. After fitting the experimental data measured by the E866 [31], E895 [32,33], E802 [34,35], NA49 [36,37], STAR [38,39,40], and ALICE Collaborations [41,42,43], we analyze the tendency of parameters.

The remainder of this paper is structured as follows. The formalism and method are shortly described in Section 2. Results and discussion are given in Section 3. In Section 4, we summarize our main observations and conclusions.

## 2. Formalism and Method

The Tsallis distribution has different forms or revisions [44,45,46,47], we have the Tsallis-like distribution of $p_{T}$ at mid-*y* to be

(1) $$\begin{array}{c} \frac{d^{2}N}{dydp_{T}}\propto \frac{dN}{dy}m_{T}{\left[1+\frac{\left(q-1\right)\left(m_{T}-\mu -m_{0}\right)}{T}\right]}^{-1/(q-1)}, \end{array}$$

where *N* denotes the number of particles,

(2) $$\begin{array}{c} m_{T}=\sqrt{p_{T}^{2}+m_{0}^{2}} \end{array}$$

can be obtained using $p_{T}$,

(3) $$\begin{array}{c} q=1+\frac{1}{n} \end{array}$$

is an entropy index that characterizes the degree of equilibrium or non-equilibrium, *n* is a parameter related to *q*, and μ is the chemical potential. In particular, in the expression of $m_{T}-\mu -m_{0}$, $m_{T}$ is simplified from $m_{T}\cosh y$ because coshy≈1 at mid-*y*.

We have the probability density function of $p_{T}$ at mid-*y* to be

(4) $$\begin{array}{c} \frac{1}{N}\frac{dN}{dp_{T}}\propto m_{T}{\left[1+\frac{\left(q-1\right)\left(m_{T}-\mu -m_{0}\right)}{T}\right]}^{-1/(q-1)}. \end{array}$$

Empirically, to fit the spectra of $p_{T}$ at mid-*y* in this work, Equation (4) can be revised as

(5) $$\begin{array}{cc} f(p_{T},T) & =Cm_{T}^{a_{0}}{\left[1+\frac{\left(q-1\right)\left(m_{T}-\mu -m_{0}\right)}{T}\right]}^{-1/(q-1)}, \end{array}$$

where *C* is the normalization constant; $a_{0}$ is a new non-dimensional parameter that describes the bending degree of the distribution in low-$p_{T}$ region ($p_{T}=0∼1$ GeV/*c*), which is introduced artificially and tested in our recent work [48,49]; and $m_{T}^{a_{0}}$ is revised from $m_{T}$ due to the introduction of the revised index $a_{0}$. Because of the limitation of the normalization, changing the bending degree in the low-$p_{T}$ region will change the slope in the high-$p_{T}$ region. Although writing $Cm_{T}^{a_{0}}$ in Equation (5) is not ideal, as it yields a fractional power unit in *C*, we have no suitable method to scale out the unit by e.g., $m_{0}$ due to the nonlinear relationship between $m_{T}$ and $m_{0}$ shown in Equation (2). In Equation (5), the other parameters such as *q* and $a_{0}$ do not appear in the function name for the purpose of convenience. In this work, we call Equation (5) the revised Tsallis-like function.

In the framework of the multisource thermal model [30], we assume that two participant partons take part in the collisions. Let $p_{t1}$ and $p_{t2}$ denote the components contributed by the first and second participant parton to $p_{T}$, respectively, where $p_{t1}$ ($p_{t2}$) is less than the transverse momentum of the participant parton. We have

(6) $$\begin{array}{c} p_{T}=\sqrt{p_{t1}^{2}+p_{t2}^{2}}, \end{array}$$

where the two components are perpendicular due to the fact that $p_{t1}$ and $p_{t2}$ are assumed to be the two components of the vector $p_{T}$. Although multiparton collisions can be important especially for central high-energy nucleus–nucleus collisions, the main contributors to particle production are still binary parton collisions, which are also the basic collision process. After all, the probability that three or more partons collide simultaneously is small. Instead, the probability of binary parton collisions is large.

In binary parton collisions, each parton, e.g., the *i*-th parton, is assumed to contribute to $p_{T}$ to obey Equation (5), where i=1 and 2. The probability density functions at mid-*y* obeyed by $p_{t1}$ and $p_{t2}$ is

(7) $$\begin{array}{cc} f_{i}\left(p_{ti},T\right) & =Cm_{ti}^{a_{0}}{\left[1+\frac{\left(q-1\right)\left(m_{ti}-\mu_{i}-m_{0i}\right)}{T}\right]}^{-1/(q-1)}, \end{array}$$

where the subscript *i* is used for the quantities related to the *i*-th parton and $m_{0i}$ is empirically the constituent mass of the considered parton. Generally, in the case of considering *u* and/or *d* quarks, we take $m_{u}=m_{d}=0.3$ GeV/$c^{2}$. It is noted that the constituent quark masses of 0.3 GeV are not incompatible with the pion and kaon masses because the collisions between the two participant quarks can produce more than one particle. The conservation of energy is satisfied in the collisions. The value of $\mu_{i}$ will be discussed at the end of this section.

Let ϕ denote the azimuthal angle of $p_{T}$ relative to $p_{t1}$. According to the works in [50,51], we have the unit normalized probability density function of $p_{T}$ and ϕ to be

(8) $$\begin{array}{cc} f_{p_{T},\phi }\left(p_{T},\phi ,T\right) & =p_{T}f_{1,2}\left(p_{t1},p_{t2},T\right)\\ \; & =p_{T}f_{1}\left(p_{t1},T\right)f_{2}\left(p_{t2},T\right)\\ \; & =p_{T}f_{1}\left(p_{T}\cos\phi ,T\right)f_{2}\left(p_{T}\sin\phi ,T\right), \end{array}$$

where $f_{1,2}\left(p_{t1},p_{t2},T\right)$ denotes the united probability density function of $p_{t1}$ and $p_{t2}$. Further, we have the probability density function of $p_{T}$ to be

(9) $$\begin{array}{cc} f_{p_{T}}\left(p_{T},T\right) & =\int_{0}^{2\pi }f_{p_{T},\phi }\left(p_{T},\phi ,T\right)d\phi \\ \; & =p_{T}\int_{0}^{2\pi }f_{1}\left(p_{T}\cos\phi ,T\right)f_{2}\left(p_{T}\sin\phi ,T\right)d\phi . \end{array}$$

**Figure 1:**
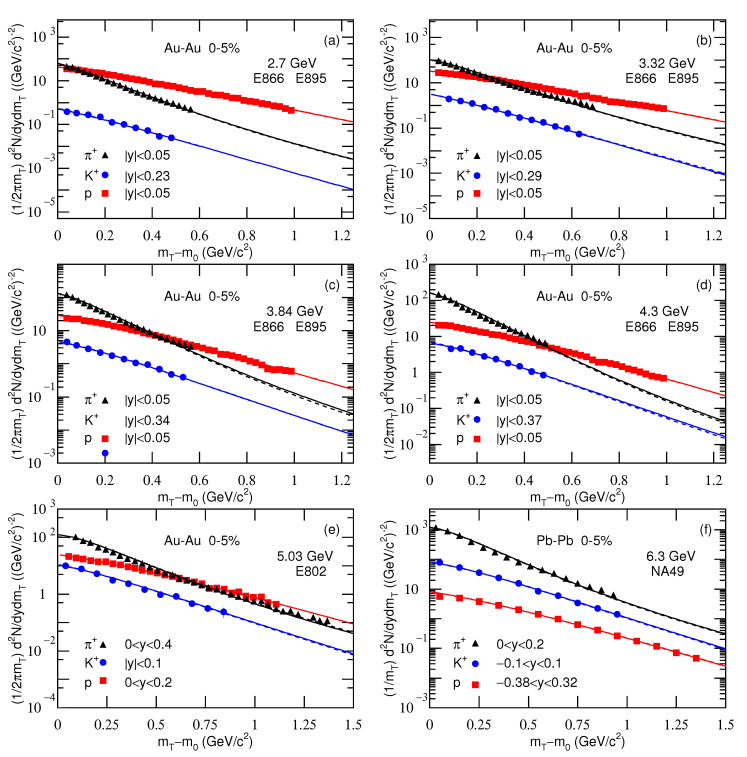
Transverse mass spectra of charged pions, kaons, and protons produced in 0–5% Au-Au collisions at $\sqrt{s_{NN}}=$ (**a**) 2.7, (**b**) 3.32, (**c**) 3.84, (**d**) 4.3, and (**e**) 5.03 GeV, and in 0–5% Pb-Pb collisions at $\sqrt{s_{NN}}=$ (**f**) 6.3 GeV. In panel (**f**), the factor 1/2π does not appear, which causes different normalization from other panels. The symbols represent the experimental data at mid-*y* measured by the E866, E895, and E802 Collaboration at the AGS [31,32,33,34,35] and by the NA49 Collaboration at the SPS [36,37]. The solid and dashed curves are our results, fitted by using Equation (10) due to Equations (7) and (9), with $\mu_{i}=0$ and $\mu_{i}=\mu_{B}/3$, respectively.

**Figure 2:**
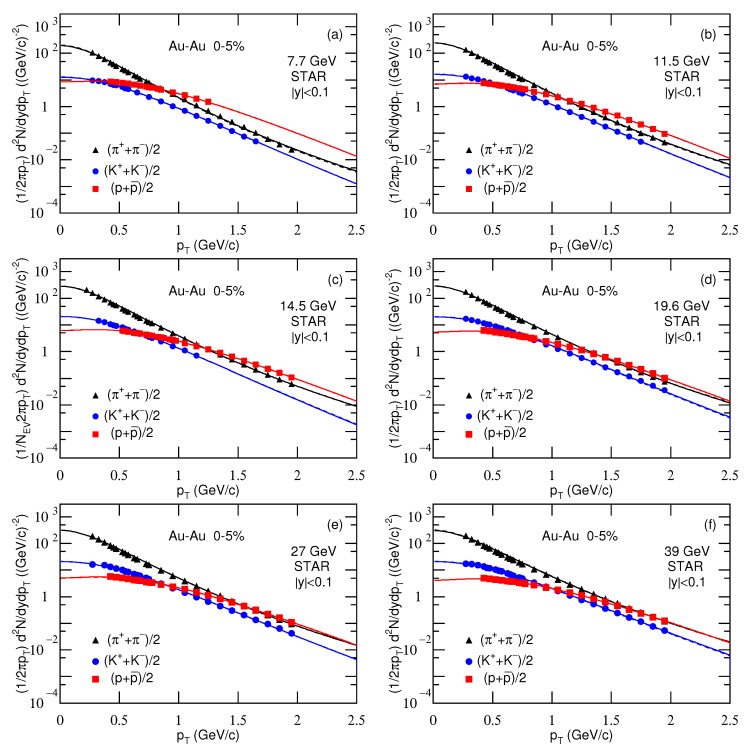
Transverse momentum spectra of charged pions, kaons, and protons produced in 0–5% Au-Au collisions at $\sqrt{s_{NN}}=$ (**a**) 7.7, (**b**) 11.5, (**c**) 14.5, (**d**) 19.6, (**e**) 27, and (**f**) 39 GeV. In panel (**c**), the factor $1/N_{EV}$, i.e., the number of events is included on the vertical axis, which can be omitted. The symbols represent the experimental data at mid-*y* measured by the STAR Collaboration at the RHIC [38,39,40]. The solid and dashed curves are our results, fitted by using Equation (10) due to Equations (7) and (9), with $\mu_{i}=0$ and $\mu_{i}=\mu_{B}/3$, respectively.

**Figure 3:**
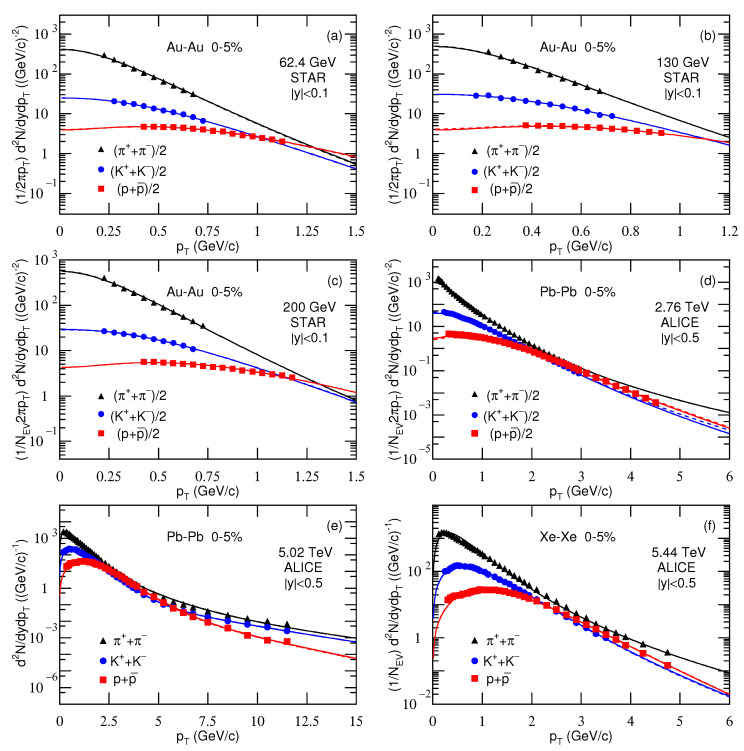
Transverse momentum spectra of charged pions, kaons, and protons produced in 0–5% Au-Au collisions at $\sqrt{s_{NN}}=$ (**a**) 62.4, (**b**) 130, and (**c**) 200 GeV; in 0–5% Pb-Pb collisions at $\sqrt{s_{NN}}=$ (**d**) 2.76 and (**e**) 5.02 TeV; and in 0–5% Xe-Xe collisions at $\sqrt{s_{NN}}=$ (**f**) 5.44 TeV. In panels (**c**,**d**,**f**), the factor $1/N_{EV}$ is included on the vertical axis, which can be omitted. In panels (**e**,**f**), the item ${\left(2\pi p_{T}\right)}^{-1}$ is not included on the vertical axis, which results in different calculation for vertical values from other panels in the normalization. The symbols represent the experimental data at mid-*y* measured by the STAR Collaboration at the RHIC [38,39,40] and by the ALICE Collaboration at the LHC [41,42,43]. The solid and dashed curves are our results, fitted by using Equation (10) due to Equations (7) and (9), with $\mu_{i}=0$ and $\mu_{i}=\mu_{B}/3$, respectively.

Equation (9) can be used to fit the $p_{T}$ spectra and obtain the parameters *T*, *q*, and $a_{0}$. In the case of fitting a wide $p_{T}$ spectrum e.g., $p_{T}>5$ GeV/*c*, Equation (9) cannot fit well the spectra in high-$p_{T}$ region. Then, we need a superposition of one Equation (9) with low *T* and another Equation (9) with high *T* to fit the whole $p_{T}$ spectrum. As will be seen in Figure 3e in the next section, the contribution fraction of the low *T* component is very large (≈99.9%). In most cases in Figure 1, Figure 2 and Figure 3, we do not need the superposition due to narrow $p_{T}$ spectra. In the case of using a two-component distribution, we have the probability density function of $p_{T}$ to be

(10) $$\begin{array}{c} f_{p_{T}}\left(p_{T}\right)=kf_{p_{T}}\left(p_{T},T_{1}\right)+\left(1-k\right)f_{p_{T}}\left(p_{T},T_{2}\right), \end{array}$$

where *k* (1−k) denotes the contribution fraction of the first (second) component and $f_{p_{T}}\left(p_{T},T_{1}\right)$ [$f_{p_{T}}\left(p_{T},T_{2}\right)$] is given by Equation (9). The second component is related to the core–corona picture as mentioned later on in detail in subsection 3.3. Correspondingly, the temperature

(11) $$\begin{array}{c} T=kT_{1}+\left(1-k\right)T_{2} \end{array}$$

is averaged by weighting the two fractions. The temperature *T* defined by Equation (11) reflects the common effective temperature of the two components which are assumed to stay in a new equilibrium in which *T* still characterizes the average kinetic energy. Similarly, the weighted average can be performed for other parameters in the two components in Equation (10).

Note that the limit of the first and second (low- and high-$p_{T}$) components is determined by a convenient treatment. Generally, the contribution fraction *k* of the first component should be taken as largely as possible. As will be seen in the next section, we take k=1 in most cases; only in Figure 3e we take k=0.999. Because the contribution fraction of the second component is zero or small enough, Equation (10) becomes Equation (9), and the weighted average of the two parameters in Equation (10) becomes the parameter in Equation (9). Because Equations (1), (4), (5), and (7) are suitable at mid-*y*, Equations (8)–(10) are also suitable at mid-*y*. In addition, the rapidity ranges quoted in the next section are narrow and around 0, though the concrete ranges are different. This means that the mentioned equations are applicable.

We would like to point out that although the model used by itself is not enough to provide information of the deconfinement phase transition from hadronic matter to QGP, the excitation function of extracted parameter is expected to show some particular tendencies. These particular tendencies include, but are not limited to, the peak and valley structures, the fast and slow variations, the positive and negative changes, etc. These particular tendencies are related to the equation of state (EOS) of the considered matter. The change of EOS reflects the possible change of interaction mechanism from hadron-dominated to parton-dominated intermediate state. Then, the deconfinement phase transition of the considered matter from hadronic matter to QGP is possibly related to the particular tendencies. It is natural that the explanations are not only for a given set of data. The present model will show a method to fit and explain the data.

To obtain $\beta_{T}$, we need to know the slope of $\langle p_{T}\rangle$ versus $\bar{m}$ in the source rest frame of the considered particle. That is, we need to calculate $\langle p_{T}\rangle$ and $\bar{m}$. According to Equation (10), we have

(12) $$\begin{array}{c} \langle p_{T}\rangle =\int_{0}^{p_{T\max}}p_{T}f_{p_{T}}\left(p_{T}\right)dp_{T} \end{array}$$

due to

(13) $$\begin{array}{c} \int_{0}^{p_{T\max}}f_{p_{T}}\left(p_{T}\right)dp_{T}=1, \end{array}$$

where $p_{T\max}$ denotes the maximum $p_{T}$.

As the mean energy, $\bar{E}=\bar{m}=\langle \sqrt{p^{2}+m_{0}^{2}}\rangle$, where *p* is the momentum of the considered particle in the source rest frame. The analytical calculation of $\bar{m}$ is complex. Instead, we can perform the calculation by the Monte Carlo method. Let $R_{1,2}$ denote random numbers distributed evenly in [0,1]. Each concrete $p_{T}$ satisfies

(14) $$\begin{array}{c} \int_{0}^{p_{T}}f_{p_{T}}\left(p_{T}^{\prime },T\right)dp_{T}^{\prime }<R_{1}<\int_{0}^{p_{T}+\delta p_{T}}f_{p_{T}}\left(p_{T}^{\prime },T\right)dp_{T}^{\prime }, \end{array}$$

where $\delta p_{T}$ denotes a small shift relative to $p_{T}$. Each concrete emission angle θ satisfies

(15) $$\begin{array}{c} \theta =2\arcsin\sqrt{R_{2}} \end{array}$$

due to the fact that the particle is assumed to be emitted isotropically in the source rest frame. Each concrete momentum *p* and energy *E* can be obtained by

(16) $$\begin{array}{c} p=p_{T}\csc\theta \end{array}$$

and

(17) $$\begin{array}{c} E=\sqrt{p^{2}+m_{0}^{2}} \end{array}$$

respectively.

After repeating calculations multiple times in the Monte Carlo method, we can obtain $\bar{E}$, that is, $\bar{m}$. Then, the slope of $\langle p_{T}\rangle$ versus $\bar{m}$ is identified as $\beta_{T}$. Meanwhile, the intercept of *T* versus $m_{0}$ is identified as $T_{0}$. Here, we emphasize that we have used the alternative method introduced in Section 1 to obtain $T_{0}$ and $\beta_{T}$.

Note that in some cases, the transverse spectra are shown in terms of $m_{T}$, but not $p_{T}$. To transform the probability density function $f_{p_{T}}\left(p_{T},T\right)$ of $p_{T}$ to the probability density function $f_{m_{T}}\left(m_{T},T\right)$ of $m_{T}$, we have the relation

(18) $$\begin{array}{c} f_{p_{T}}\left(p_{T},T\right)|dp_{T}|=f_{m_{T}}\left(m_{T},T\right)\left|dm_{T}\right|. \end{array}$$

Then, we have

(19) $$\begin{array}{c} f_{m_{T}}\left(m_{T},T\right)=\frac{m_{T}}{\sqrt{m_{T}^{2}-m_{0}^{2}}}f_{p_{T}}\left(\sqrt{m_{T}^{2}-m_{0}^{2}},T\right) \end{array}$$

due to Equation (2). Using the parameters from $m_{T}$ spectra, we may also obtain $T_{0}$, $\langle p_{T}\rangle$, $\bar{m}$, and $\beta_{T}$.

We now discuss the chemical potential $\mu_{i}$ of the *i*-th parton. Generally, the chemical potential μ of a particle obviously affects the particle production at low energy [52,53,54,55,56,57,58]. For baryons (mostly protons and neutrons), the chemical potential $\mu_{B}$ related to collision energy $\sqrt{s_{NN}}$ is empirically given by

(20) $$\begin{array}{c} \mu_{B}=\frac{1.303}{1+0.286\sqrt{s_{NN}}}, \end{array}$$

where both $\mu_{B}$ and $\sqrt{s_{NN}}$ are in the units of GeV [59,60,61]. According to the authors of [52], we have $\mu_{u}=\mu_{d}=\mu_{B}/3$ because a proton or neutron is consists of three u/d quarks (i.e., uud or udd).

## 3. Results and Discussion

### 3.1. Comparison with Data and Tendencies of Free Parameters

Figure 1, Figure 2 and Figure 3 present the transverse momentum $p_{T}$ (transverse mass $m_{T}$) spectra, ${\left(2\pi p_{T}\right)}^{-1}d^{2}N/dydp_{T}$$\left[{\left(2\pi m_{T}\right)}^{-1}d^{2}N/dydm_{T}\right]$, of charged pions, kaons, and protons produced in 0–5% Au-Au, Pb-Pb, and Xe-Xe collisions at different $\sqrt{s_{NN}}$. The collision types, particle types, mid-*y* ranges, centrality classes, and $\sqrt{s_{NN}}$ are marked in the panels. The symbols represent the experimental data measured by different collaborations. The solid and dashed curves are our results, fitted by using Equation (10) due to Equations (7) and (9), with $\mu_{i}=0$ and $\mu_{i}=\mu_{B}/3$, respectively. In the process of fitting the data, we determine the best parameters by the method of least squares. The experimental uncertainties used in calculating the $\chi^{2}$ are obtained by the root sum square of the statistical uncertainties and the systematic uncertainties. The parameters that minimize the $\chi^{2}$ are the best parameters. The errors of parameters are obtained by the statistical simulation method [62,63], which uses the same algorithm as usual, if not the same Code, in which the errors are also extracted from variations of $\chi^{2}$. The values of $T_{1}$, $T_{2}$, *k*, *q*, and $a_{0}$ are listed in Table 1 and Table 2 with the normalization constant ($N_{0}$), $\chi^{2}$, and the number of degree of freedom (ndof), or explained in the caption of Table 1.

In a few cases, the values of $\chi^{2}$/ndof are very large (5–10 or above), which means “bad” fit to the data. In most cases, the fits are good due to small $\chi^{2}$/ndof which is around 1. To avoid possible wrong interpretation with this result, the number of “bad” fits are limited to be much smaller than that of good fits, for example, 1 to 5 or more strict such as 1 to 10. Meanwhile, we should also use other method to check the quality of fits. In fact, we have also calculated the p-values in the Pearson method. It is shown that all p-values are less than $3\times 10^{-7}$. These p-values corresponds approximately to the Bayes factor being above 100 and to the confidence degree of 99.99994% at around 5 standard deviations (5σ) of the statistical significance. This means that the model function is in agreement with the data very well. To say the least, most fits are acceptable.

Note that we will use a set of pion, kaon, and proton spectra to extract $T_{0}$ and $\beta_{T}$ in Section 3.2. For energies in the few GeV range, the spectra of some negative particles are not available in the literature. Therefore, we have to give up to analyze all the negative particle spectra in Figure 1. In our recent work [28], the positive and partial negative particle spectra were analyzed by the standard distribution. The tendencies of parameters are approximately independent of isospin, if not the same for different isospins.

One can see from Figure 1, Figure 2 and Figure 3 and Table 1 and Table 2 that Equation (10) approximately describes the considered experimental data. For all energies and particles, $T_{1}$ and $T_{2}$ are identical except for the 5.02 TeV Pb-Pb data from ALICE. This means that none of the spectra have a wide enough range to determine the second component except the data at 5.02 TeV. The two-component fit is only really used at 5.02 TeV. In the high-$p_{T}$ region, the hard scattering process which is described by the second component in Equation (10) contributes totally. However, in the case of using the two-component function, *k* (=0.999) is very close to 1, which implies that the contribution of the second component is negligible. In fact, the second component contributes to the spectrum in high-$p_{T}$ region with small fraction, which does not affect significantly the extraction of parameters. Instead, the parameters are determined mainly by the spectrum in low-$p_{T}$ region.

Although the contribution fraction of the second component is very small, the spectra with a wide $p_{T}$ range on Figure 3e are well fit using the two components, this means increasing the number of parameters compared with just Tsallis function. Generally, the spectrum shapes of different particles are different. However, we may use the same function with different parameters and normalization constants to fit them uniformly. In some cases, the spectrum forms are different. We need to consider corresponding normalization treatments so that the fitting function and the data are compatible and concordant.

The value of $\mu_{i}$ affects mainly the parameters at below dozens of GeV. Although $\mu_{i}=0$ is not justified at lower energies, we present the results with $\mu_{i}=0$ for comparison with $\mu_{i}=\mu_{B}/3$ so that we can have a quantitative understanding on the influence of $\mu_{i}$. Note that $\mu_{i}$ is only for $\mu_{u}$ and $\mu_{d}$, that is $\mu_{u}=\mu_{d}=\mu_{B}/3$. For pions, we have $\mu_{\pi }=\mu_{u}+\mu_{d}=2\mu_{B}/3$. For kaons, we have no suitable expression because the chemical potential $\mu_{s}$ for *s* quark is not available here. Generally, $\mu_{s}>\mu_{u}$. Therefore, $\mu_{K}=\mu_{u}+\mu_{s}>\mu_{\pi }$.

As a function with wide applications, the Tsallis distribution can describe in fact the spectra presented in Figure 1, Figure 2 and Figure 3 in most cases, though the values of parameters may be changed. However, to extract some information at the parton level, we have regarded the revised Tsallis-like function (Equation (7)) as the components of $p_{T}$ contributed by the participant partons. The value of $p_{T}$ is then taken to be the root sum square of the components. In the present work, we have considered two participant partons and two components. This treatment can be extended to three and more participant partons and their components. In the case of the analytical expression for more components becoming difficult, we may use the Monte Carlo method to obtain the components, and $p_{T}$ is also the root sum square of the components. Then, the distribution of $p_{T}$ is obtained by the statistical method.

**Figure 4:**
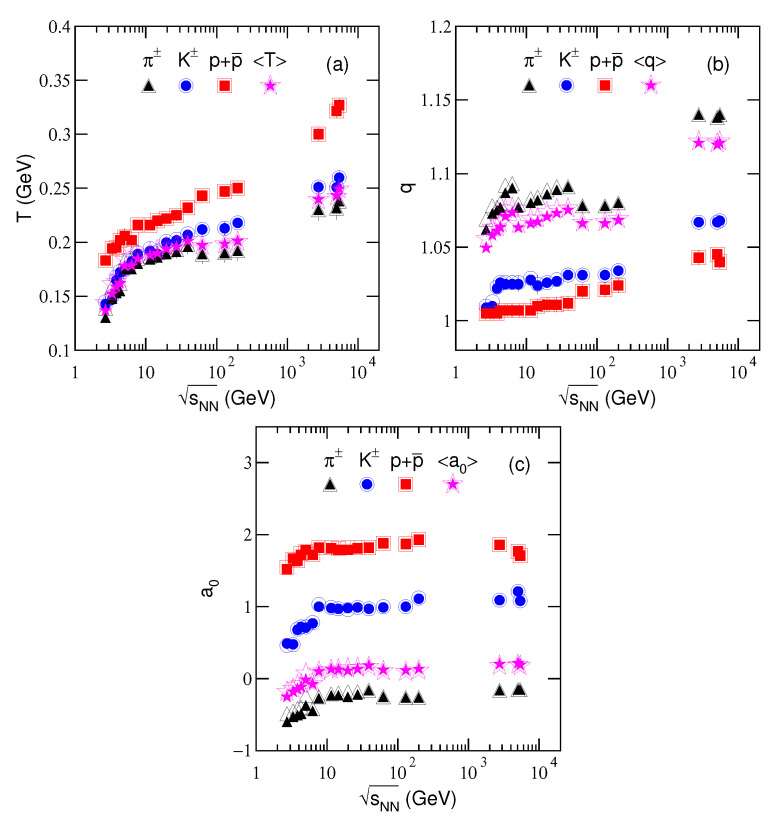
Dependences of (**a**) effective temperature *T*, (**b**) entropy index *q*, and (**c**) revised index $a_{0}$ on energy $\sqrt{s_{NN}}$, where the closed and open symbols are cited from Table 1 and Table 2 which are obtained from the fittings with $\mu_{i}=0$ (solid curves) and $\mu_{i}=\mu_{B}/3$ (dashed curves) in Figure 1, Figure 2 and Figure 3, respectively. The triangles, circles, squares, and pentagrams represent the results for charged pions, kaons, protons, and the average by weighting different yields, respectively.

To study the changing trends of the free parameters, Figure 4 shows the dependences of (a) effective temperature *T*, (b) entropy index *q*, and (c) revised index $a_{0}$ on collision energy $\sqrt{s_{NN}}$, where the closed and open symbols are cited from Table 1 and Table 2 which are obtained from the fittings with $\mu_{i}=0$ (solid curves) and $\mu_{i}=\mu_{B}/3$ (dashed curves) in Figure 1, Figure 2 and Figure 3, respectively. The triangles, circles, squares, and pentagrams represent the results for charged pions, kaons, protons, and the average by weighting different yields, respectively. Because the errors of parameters are very small, the error bars in the plots are invisible. One can see from Figure 4 that *T* increases significantly, *q* increases slowly, and $a_{0}$ increase quickly from ≈3 to ≈10 GeV (exactly from 2.7 to 7.7 GeV) and then changes slowly at above 10 GeV, except for a large increase (≈50%) at the maximum energy, with the increase of $\ln(\sqrt{s_{NN}})$. These parameters also show their dependences on particle mass $m_{0}$: With the increase of $m_{0}$, *T* and $a_{0}$ increase and *q* decreases significantly. Indeed, $\mu_{i}$ affects only the parameters at the lower energies (below dozens of GeV), but not higher energy.

The behavior of excitation function of *T* will be discussed as that of $T_{0}$ in the next subsection. The large fluctuations of *q* for pions are caused by the large influence of strong decay from high-mass resonance and weak decay from heavy flavor hadrons. For light particles such as pions, the influence and then the fluctuations are large; while for relative heavy particles such as kaons and protons, the influence and then the fluctuations are small. No matter how large the fluctuations are, the values of *q* are close to 1.

As we mentioned in the above section, the entropy index *q* reflects the degree of equilibrium or non-equilibrium of collision system. Usually, q=1 corresponds to an ideal equilibrium state and q≫1 means a non-equilibrium state. The present work shows that *q* is very close to 1 which means that the system stays in the equilibrium state. Generally, the equilibrium is relative. For the case of non-equilibrium, we may use the concept of local equilibrium. If *q* is not too large, for example, q≤1.25 or n≥4, the collision system is still in equilibrium or local equilibrium [45,64]. In particular, the system is closer to the equilibrium when it emits protons at lower energy, comparing with pions and kaons at higher energy. The reason is that most protons came from the participant nuclei directly. They have enough time to reach to the equilibrium in the evolution. At lower energy, the system is closer to the equilibrium because the evolution is slower and the system has more time to result in the equilibrium. From the initial collisions to kinetic freeze-out, the evolution time is very short. The lower the collision energy is, the longer the evolution time is.

The values of $a_{0}$ for the spectra of charged pions, kaons, and protons at above 10 GeV are approximately 0.75, 1, and 1.8, respectively, which drop obviously for pions and kaons at lower energy due to the hadronic phase. In addition, due to the existence of participant protons in both the hadronic and QGP phases, the energy dependence of $a_{0}$ for protons is not obvious. Although it is hard to explain exactly the physical meaning of $a_{0}$, we emphasize here that it shows the bending degree of the spectrum in low-$p_{T}$ region [48,49] and affects the slopes in high-$p_{T}$ region due to the limitation of normalization. A large bending degree means a large slope change. In fact, $a_{0}$ is empirically related to the contributions of strong decay from high-mass resonance and weak decay from heavy flavor hadrons. This is because that $a_{0}$ affects mainly the spectra in low-$p_{T}$ region which is just the main contribution region of the two decays.

One can see that the values of *q* and $a_{0}$ change drastically with particle species. This is an evidence of mass-dependent differential kinetic freeze-out scenario [26]. The massive particles emit earlier than light particles in the system evolution. The earlier emission is caused due to the fact that the massive particles are left behind in the evolution process, but not their quicker thermal and flow motion. In fact, the massive particles have no quicker thermal and flow motion due to larger mass. Instead, light particles have quicker thermal and flow motion and longer evolution time. Finally, light particles reach larger space at the stage of kinetic freeze-out.

The influence of $\mu_{i}$ on *q* and $a_{0}$ is very small. Although the prefactor $a_{0}$ can come from the Cooper–Frye term (and/or a kind of saddlepoint integration) as discussed, e.g., in [65,66], it is a fit parameter in this work. As an average over pions, kaons, and protons, $\langle a_{0}\rangle$ is nearly independent of $\sqrt{s_{NN}}$ at above 10 GeV. As $\sqrt{s_{NN}}$ increasing from ≈3 to ≈10 GeV, the increase of $\langle a_{0}\rangle$ shows different collision mechanisms comparing with that at above 10 GeV. Our recent work [67] shows that the energy ≈10 GeV discussed above is exactly 7.7 GeV.

### 3.2. Derived Parameters and Their Tendencies

As we know, the effective temperature *T* contains the contributions of the thermal motions and flow effect [68]. The thermal motion can be described by the kinetic freeze-out temperature $T_{0}$, and the flow effect can be described by the transverse flow velocity $\beta_{T}$. To obtain the values of $T_{0}$ and $\beta_{T}$, we analyze the values of *T* presented in Table 1 and Table 2, and calculate $\langle p_{T}\rangle$ and $\bar{m}$ based on the values of parameters listed in Table 1 and Table 2. In the calculation performed from $p_{T}$ to $\langle p_{T}\rangle$ and $\bar{m}$ by the Monte Carlo method, as in [24,25,26], an isotropic assumption in the rest frame of emission source is used.

**Figure 5:**
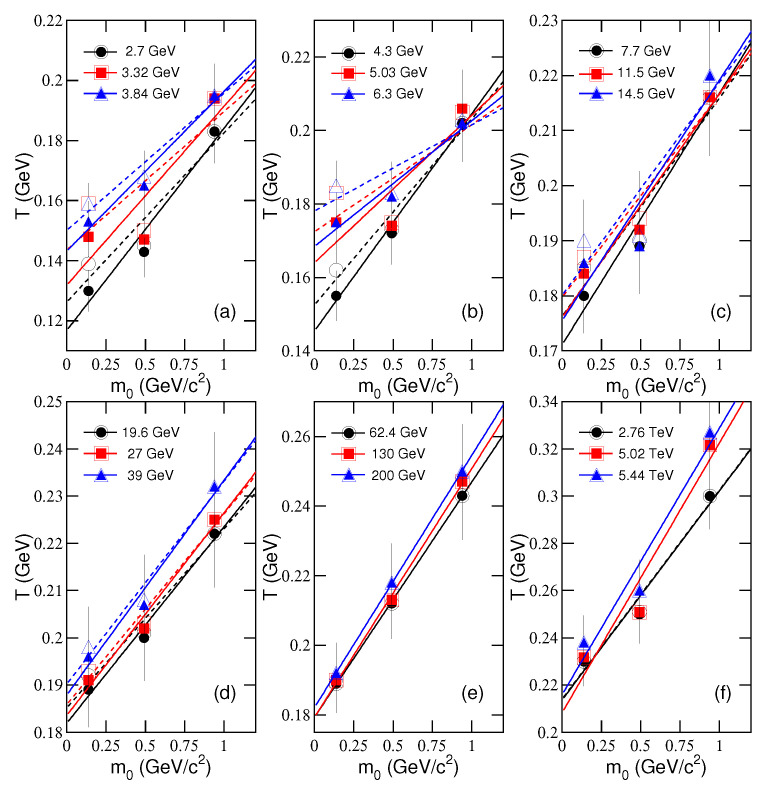
Dependences of *T* on $m_{0}$. Different symbols represent the results from identified particles produced in central AA collisions at different energies shown in panels (**a**)–(**f**). The lines are the results fitted by the least square method, where the intercepts are regarded as $T_{0}$. The closed and open symbols (the solid and dashed curves) correspond to the results for $\mu_{i}=0$ and $\mu_{i}=\mu_{B}/3$ respectively.

**Figure 6:**
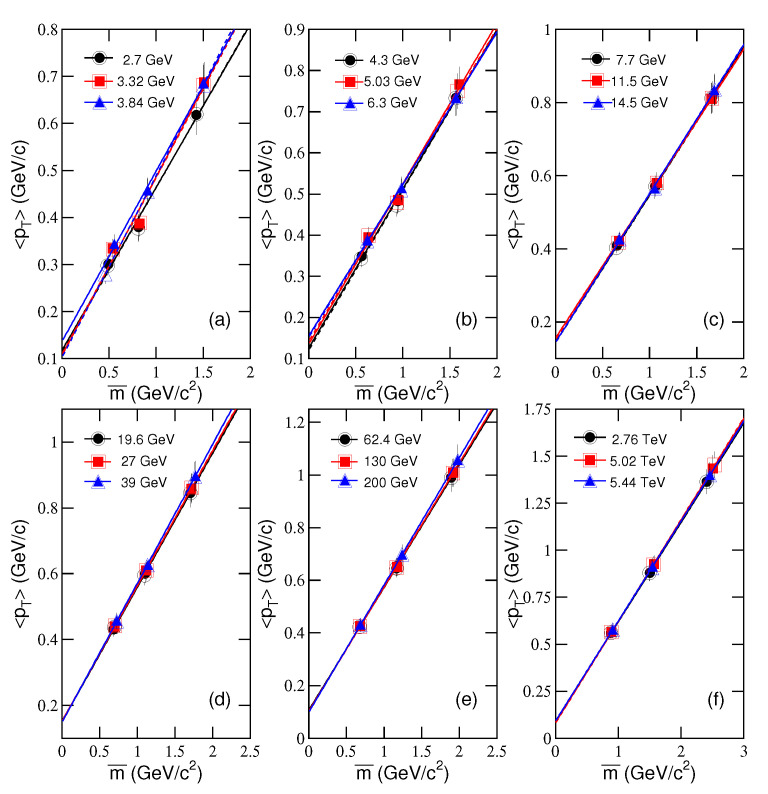
Different symbols represent the results from identified particles produced in central AA collisions at different energies shown in panels (**a**)–(**f**). Same as for Figure 5, but showing the dependences of $\langle p_{T}\rangle$ on $\bar{m}$. The lines are the results fitted by the least square method, where the slopes are regarded as $\beta_{T}$.

Figure 5a–f shows the relationship of *T* and $m_{0}$, determined fitting AA collision systems by our model. Figure 6a–f shows the relationship of $\langle p_{T}\rangle$ and $\bar{m}$, correspondingly. Different symbols represent the values from central AA collisions at different $\sqrt{s_{NN}}$. The symbols in Figure 5a–f represent the values of *T* for different $m_{0}$. The symbols in Figure 6a–f represent the values of $\langle p_{T}\rangle$ for different $\bar{m}$.

We noted that in Figure 5b, *T* increases with the energy from 4.3 to 6.3 GeV for the emission of pions and not for protons, while in the case of 2.76–5.44 TeV in Figure 5f, *T* increases for the emission of protons and not for pions. This discrepancy also appears when narrow energy ranges are fitted in experiments, though $\langle p_{T}\rangle$ should increase for all particle species as a function of $\sqrt{s_{NN}}$. We may explain this as the fluctuations. It is expected that *T* for emissions of both pions and protons show the same or similar behavior with the energy in a wide range.

It can be seen that the mentioned relationships show nearly linear tendencies in most cases. The lines in Figure 5 and Figure 6 are the results fitted by the least square method, where the solid and dashed lines correspond to the results for $\mu_{i}=0$ and $\mu_{i}=\mu_{B}/3$, respectively. The values of intercepts, slopes, and $\chi^{2}$ are listed in Table 3 and Table 4. One can see that, in most cases, the mentioned relations are described by a linear function. In particular, the intercepts in Figure 5a–f are regarded as $T_{0}$, and the slopes in Figure 6a–f are regarded as $\beta_{T}$, as what we discussed above in the alternative method. Because different “thermometers” are used, $T_{0}$ extracted from the intercept exceeds (is not in agreement with) the transition temperature which is independently determined by lattice QCD to be around 155 MeV. To compare the two temperatures, we need a transform equation or relation which is not available at present and we will discuss it later.

It is noted that the above argument on $T_{0}$ and $\beta_{T}$ is based usually on exact hydrodynamic calculations, as, e.g., given in [17,65,69,70,71,72]. However, in these cases, usually *T* is extracted, and then some $T=T_{0}+m_{0}{\langle u_{t}\rangle }^{2}$ like correspondence is derived (where instead of $m_{0}$, also energy or average energy could stand, depending on the calculation). Here, as we know, $\langle u_{t}\rangle$ is related but not equal to $\beta_{T}$, as discussed in the mentioned literature. Therefore, we give up to use $\langle u_{t}\rangle$ as $\beta_{T}$ in this work.

We think that $T_{0}$ can be also obtained from $\langle p_{T}\rangle$, and $\beta_{T}$ can be also obtained from *T*. However, the relations between $T_{0}$ and $\langle p_{T}\rangle$, as well as $\beta_{T}$ and *T*, are not clear. Generally, the parameters $T_{0}$ and $\beta_{T}$ are model-dependent. In other models, such as the blast-wave model [17,18,19,20,21], $T_{0}$ and $\beta_{T}$ can be obtained conveniently. The two treatments show similar tendencies of parameters on $\sqrt{s_{NN}}$ and event centrality, if we also consider the flow effect in small system or peripheral AA collisions [73,74] in the blast-wave model.

**Figure 7:**
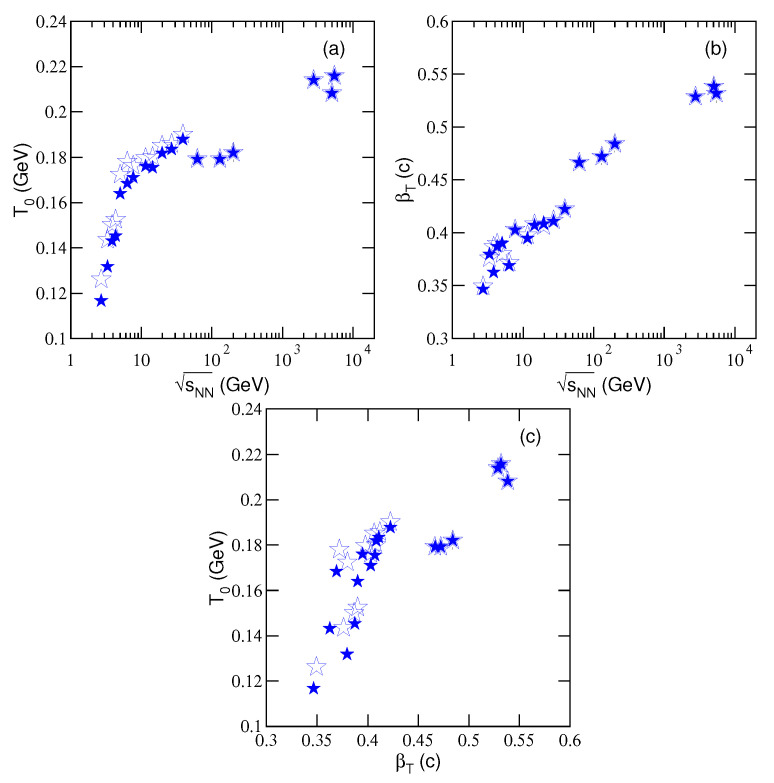
Dependences of (**a**) $T_{0}$ on $\sqrt{s_{NN}}$, (**b**) $\beta_{T}$ on $\sqrt{s_{NN}}$, and (**c**) $T_{0}$ on $\beta_{T}$. The parameter values are obtained from Table 3 and Table 4, which are from the linear fittings in Figure 5 and Figure 6.

In order to more clearly see the tendencies of $T_{0}$ and $\beta_{T}$, we show the dependences of $T_{0}$ on $\sqrt{s_{NN}}$, $\beta_{T}$ on $\sqrt{s_{NN}}$, and $T_{0}$ on $\beta_{T}$ in Figure 7a–c, respectively. One can see that the two parameters increase quickly from ≈3 to ≈10 GeV and then slowly at above 10 GeV with the increase of $\sqrt{s_{NN}}$ in general. There is a plateau from near 10 GeV to 200 GeV. In particular, $T_{0}$ increases with $\beta_{T}$ due to the fact that both of them increase with $\sqrt{s_{NN}}$. These incremental tendencies show that, in the stage of kinetic freeze-out, the degrees of excitation and expansion of the system increase with increasing $\sqrt{s_{NN}}$. These results are partly in agreement with the blast-wave model which shows decreasing tendency for $T_{0}$ and increasing tendency for $\beta_{T}$ with increasing $\sqrt{s_{NN}}$ from the RHIC [40] to LHC [41] because different partial $p_{T}$ ranges in the data are considered for different particles, while this work uses the $p_{T}$ range as wide as the data. The chemical potential shows obvious influence on $T_{0}$ at the lower energies (below dozens of GeV). After considering the chemical potential, the plateau in the excitation function of $T_{0}$ becomes more obvious.

With the increase of $\sqrt{s_{NN}}$, the fact that the values of $T_{0}$ and $\beta_{T}$ increase quickly from ≈3 to ≈10 GeV and then slowly at above 10 GeV implies that there are different collision mechanisms in the two energy ranges. In AA collisions, if the baryon-dominated effect plays a more important role at below 10 GeV [75], the meson-dominated effect should play a more important role at above 10 GeV. In the baryon-dominated case, less energies are deposited in the system, and then the system has low excitation degree and temperature. In the meson-dominated case, the situation is opposite. Indeed, ≈10 GeV is a particular energy which should be paid more attention. It seems that the onset energy of deconfinement phase transition from hadronic matter to QGP is possibly 10 GeV or slightly lower (e.g., 7.7 GeV [67]).

If we regard the plateau from near 10 to 200 GeV in the excitation functions of $T_{0}$ and $\beta_{T}$ as a reflection of the formation of QGP liquid drop, the quick increase of $T_{0}$ and $\beta_{T}$ at the LHC is a reflection of higher temperature QGP liquid drop due to larger energy deposition. At the LHC, the higher collision energy should create larger energy density and blast wave, and then higher $T_{0}$ and $\beta_{T}$. Although any temperature needs to be bound by the phase transition on one side and free streaming on the other side, larger energy deposition at the LHC may heat the system to a higher temperature even the phase transition temperatures at the LHC and RHIC are the same. Both the formed QGP and hadronized products are also possible to be heated to higher temperature.

Although we mentioned that the plateau apparent in $T_{0}$ versus $\sqrt{s_{NN}}$ is possibly connected to the onset of deconfinement, the temperature measured in this work is connected only to $T_{0}$ which is usually much smaller than the quark-hadron transition temperature. Because the collision process is very complex, the $\sqrt{s_{NN}}$ dependence of $T_{0}$ reflects only partial properties of the phase structure of a quark medium. To make a determined conclusion, we may connect to the dynamics of the hadron gas. This topic is beyond the focus of the present work and will not be discussed further here.

We would like to point out that, in the last three paragraphs mentioned above, the discussions on the excitation function of $T_{0}$ presented in Figure 7a are also suitable to the excitation function of *T* presented in Figure 4a, though the effect of flow is not excluded from Figure 4a. Because the quality of fits is not sufficient in a few cases, our main conclusion that the rise of temperature below 10 GeV suggests that a deconfinement of hadronic matter to QGP is weak. The information of phase transition happened at higher temperatures and near the chemical freeze-out may be reflected at the kinetic freeze-out of a hadronic system. The plateau structure appeared in the excitation function $T_{0}$ is expected to relate to the phase transition, though this relation is not clear at present. Other works related to this issue are needed to make a strong conclusion. In other words, to conclude the onset of deconfinement just from the quality of some fits is a loose interpretation. More investigations are needed and also comparison with other findings. This issue is beyond the scope of this analysis.

### 3.3. Further Discussion

The model presented in the analysis can be regarded as a “thermometer” to measure temperatures and other parameters at different energies. Then, the related excitation functions can be obtained, and the differences from the transition around critical point and other energies can be seen. Different models can be regarded as different “thermometers”. The temperatures measured by different “thermometers” have to be unified so that one can give a comparison. If we regard the phase transition temperature determined by lattice QCD as the standard one, the values of $T_{0}$ obtained in this paper should be revised to fit the standard temperature. However, this revision is not available for us at present due to many uncertain factors. In fact, we try to focus on the “plateau” in the energy dependence of $T_{0}$, but not on the $T_{0}$ values themselves.

In addition, the model assumes the contributions from two participant partons in the framework of multisource thermal model [30]. In pp collisions, one can see the point of a hard scattering between two partons and look at the high $p_{T}$ particle productions or other observations. However, even in pp collisions there are underlying events, multiple-parton interactions, etc. Further, the data used in this analysis are from central AA collisions, where hundreds and thousands of hadrons are produced. Although many partons take part in the collisions, only a given two-parton process plays main role in the production of a given set of particles. Many two-parton processes exist in the collisions. Using a model inspired by two participant partons is reasonable.

Of course, one may also expect that the production of many particles can result from three or more partons. If necessary, we may extend the picture of two participant partons to that of three or multiple participant partons [30] if we regard $p_{T}$ of identified particle as the root sum square of the transverse momenta of three or multiple participant partons. It is just that the picture of two participant partons is enough for the production of single particle in this analysis. Besides, we did not try to distinguish between local thermalization of a two-parton process. Instead, we regard the whole system as the same temperature, though which is mass dependent.

The present work is different from the quark coalescence model [66,76,77,78,79,80], though both the models have used the thermalization and statistics. In particular, the quark coalescence model describes classically mesonic prehadrons as quark–anti-quark clusters, and baryonic ones composed from three quarks. The present work describes both mesons and baryons as the products of two participant partons which are regarded as two energy sources.

The assumption of two participant partons discussed in the present work does not mean that the particles considered directly stem from two initial partons of the incoming nuclei. In fact, we assume the two participant partons from the violent collision system in which there is rescattering, recombination, or coalescence. The two participant partons are only regarded as two energy sources to produce a considered particle, whether it is a meson, baryon, or even a lepton [48,49]. The present work treats uniformly the production of final-state particles from the viewpoint of participant energy sources, but not the quark composition of the considered particles [66,76,77,78,79,80].

In the two-component distribution (Equation (10)), the first component contributed by the soft excitation process is from the sea quarks. The second component contributed by the hard scattering process is from the valence quarks. This explanation is different from the Werner’s picture on core–corona separation [81,82,83,84], in which core and corona are simply defined by the density of partons in a particular area of phase or coordinate-space and they distinguish between thermal and non-thermal particle production. This could also be a two-component fit based on the Tsallis function, but its relation to the two-parton dynamics pushed here is not clear. Anyhow, it is possible that the two processes can be described by a uniform method [48,49], though different functions and algorithms are used.

Although there were many papers in the past that have studied the identified particle spectra in high-energy collisions, both experimentally and phenomenologically, this work shows a new way to systemize the experimental data in AA collisions over a wide energy range from 2.7 GeV to 5.44 TeV at the parton level. We emphasize that, in this work, we have analyzed the particle $p_{T}$ as the root sum square of transverse momenta $p_{t1}$ and $p_{t2}$ of two participant partons. That is, the relation of $p_{T}=\sqrt{p_{t1}^{2}+p_{t2}^{2}}$ is used. While, in our recent work [48,49], the relation of $p_{T}=p_{t1}+p_{t2}$ is used, which is considered from energy relation at mid-*y* for massless particle. The scenarios used in this work and our recent work are different. Based on our analyses, it is hard to judge which scenario is more reasonable.

Through the analysis of the data, we have obtained the excitation functions of some quantities, such as *T* and its weighted average 〈T〉, $T_{0}$ and its weighted average $\langle T_{0}\rangle$, $\beta_{T}$ and its weighted average $\langle \beta_{T}\rangle$, *q* and its weighted average 〈q〉, as well as $a_{0}$ and its weighted average $\langle a_{0}\rangle$. These excitation functions all show some specific laws as $\sqrt{s_{NN}}$ increases. Although the conclusion on “onset of deconfinement” or QCD phase transition is indicated around 10 GeV or below is possibly over-interpreting the data and only using the blast-wave or Tsallis-like model is clearly not enough, the sudden change in the slope in the excitation function of $T_{0}$ is worthy of attention.

## 4. Summary and Conclusions

We summarize here our main observations and conclusions.

(a) The transverse momentum (mass) spectra of charged pions, kaons, and protons produced at mid-rapidity in central AA (Au-Au, Pb-Pb, and Xe-Xe) collisions over an energy range from 2.7 GeV to 5.44 TeV have been analyzed in this work. The experimental data measured by several collaborations are fitted satisfactorily in the framework of multisource thermal model in which the transverse momentum of identified particle is regarded as the root sum square of transverse momenta of two participant partons, where the latter obeys the revised Tsallis-like function. This treatment for the spectra of transverse momenta is novel and successful. The excitation functions of parameters such as the effective temperature, entropy index, revised index, kinetic freeze-out temperature, and transverse flow velocity are obtained. The chemical potential has obvious influence on the excitation function of kinetic freeze-out temperature at lower energy.

(b) With increasing collision energy, the entropy index increases slowly, and the revised index increases quickly and then changes slowly except for a large increase at the LHC. With increasing the particle mass, the entropy index decreases and the revised index increases obviously. The collision system discussed in this work stays approximately in the equilibrium state, and some functions based on the assumption of equilibrium can be used. The system is closer to the equilibrium state when it emits protons at lower energy, comparing with pions and kaons at higher energy. The revised index describes the bending degrees of the spectra in very low transverse momentum region. Its values for the spectra of charged pions, kaons, and protons are approximately 0.75, 1, and 1.8, respectively, at above 10 GeV and drop obviously at below 10 GeV.

(c) With increasing collision energy, the effective temperature increases clearly and monotonously, and the kinetic freeze-out temperature and transverse flow velocity increase quickly from ≈3 to ≈10 GeV and then slowly at above 10 GeV. There is a plateau from near 10 GeV to 200 GeV in the excitation functions of the latter pair. The onset energy of deconfinement phase transition from hadronic matter to QGP is connected to the special changes of excitation function of kinetic freeze-out temperature and possibly 10 GeV or slightly lower. If the plateau at the RHIC is regarded as a reflection of the formation of QGP liquid drop, the following quick increase of the excitation functions at the LHC is a reflection of higher temperature QGP liquid drop due to larger energy deposition. At kinetic freeze-out, the temperature and expansion velocity of the system increase with increasing the energy from the RHIC to LHC.

## Figures and Tables

**Table 1:** Values of free parameters ($T_{1}$, $T_{2}$, *q*, and $a_{0}$), normalization constant ($N_{0}$), $\chi^{2}$, and ndof corresponding to the solid curves in Figure 1, Figure 2 and Figure 3 in which the data are measured in special conditions (mid-*y* ranges and energies) by different collaborations, where $T_{2}$ is not available in most cases because k=1. In a few cases (at $\sqrt{s_{NN}}=5.02$ TeV), $T_{2}$ is available in the next line, where k=0.999±0.001, which is not listed in the table.

Collab.	$\sqrt{s_{\mathrm{NN}}}$ (GeV)	Particle	$T_{1}$,$T_{2}$ (MeV)	*q*	$a_{0}$	$N_{0}$	$\chi^{2}$/ndof
E866/E895 Au-Au	2.7	$\pi^{+}$	130±4	1.062±0.003	−0.60±0.01	12±2	11.87/19
		$K^{+}$	143±7	1.009±0.004	0.49±0.01	0.054±0.002	3.61/6
		*p*	183±4	1.005±0.001	1.52±0.01	75±6	153.83/36
	3.32	$\pi^{+}$	148±4	1.073±0.003	−0.53±0.01	28±2	56.96/24
		$K^{+}$	147±6	1.010±0.003	0.48±0.02	2.14±0.01	2.23/8
		*p*	194±5	1.005±0.002	1.67±0.01	69±3	237.28/36
	3.84	$\pi^{+}$	153±4	1.075±0.003	−0.51±0.02	37±6	34.34/19
		$K^{+}$	165±8	1.022±0.005	0.68±0.02	4.52±0.01	0.92/7
		*p*	195±5	1.005±0.002	1.64±0.02	61±5	308.11/36
	4.3	$\pi^{+}$	155±6	1.077±0.003	−0.49±0.02	46±9	47.97/16
		$K^{+}$	172±10	1.026±0.002	0.72±0.02	7.17±0.02	0.62/5
		*p*	202±7	1.007±0.003	1.72±0.02	59±9	75.53/36
E802 Au-Au	5.03	$\pi^{+}$	175±4	1.087±0.001	−0.37±0.01	54±6	129.94/30
		$K^{+}$	174±6	1.025±0.001	0.71±0.01	12±3	5.29/7
		*p*	206±7	1.007±0.003	1.79±0.03	62±5	47.59/25
NA49 Pb-Pb	6.3	$\pi^{+}$	175±4	1.090±0.001	−0.45±0.02	74±6	314.01/12
		$K^{+}$	182±6	1.025±0.002	0.77±0.01	100±2	41.92/6
		*p*	202±7	1.007±0.003	1.72±0.03	20±1	6.99/10
STAR Au-Au	7.7	$(\pi^{+}+\pi^{-})/2$	180±7	1.077±0.001	−0.27±0.01	90±2	42.38/22
		$(K^{+}+K^{-})/2$	189±9	1.025±0.005	1.00±0.01	14±3	3.02/16
		$(p+\bar{p})/2$	216±10	1.007±0.002	1.82±0.01	27±1	0.95/11
	11.5	$(\pi^{+}+\pi^{-})/2$	184±7	1.080±0.001	−0.23±0.01	120±5	44.84/22
		$(K^{+}+K^{-})/2$	192±9	1.028±0.003	0.98±0.01	19±3	1.07/19
		$(p+\bar{p})/2$	216±11	1.007±0.001	1.80±0.01	23±1	1.38/19
	14.5	$(\pi^{+}+\pi^{-})/2$	186±7	1.082±0.001	−0.23±0.02	142±9	4.09/24
		$(K^{+}+K^{-})/2$	189±9	1.024±0.006	0.97±0.01	22±3	0.84/14
		$(p+\bar{p})/2$	220±12	1.010±0.001	1.80±0.01	21±1	0.28/21
	19.6	$(\pi^{+}+\pi^{-})/2$	189±8	1.086±0.001	−0.25±0.03	150±6	32.66/21
		$(K^{+}+K^{-})/2$	200±9	1.026±0.003	0.98±0.01	24±4	19.01/22
		$(p+\bar{p})/2$	222±11	1.011±0.001	1.80±0.02	19±1	2.20/18
	27	$(\pi^{+}+\pi^{-})/2$	191±8	1.089±0.001	−0.22±0.01	164±6	27.71/21
		$(K^{+}+K^{-})/2$	202±9	1.027±0.003	0.99±0.01	26±3	10.49/20
		$(p+\bar{p})/2$	225±11	1.011±0.002	1.81±0.02	19±1	4.56/18
	39	$(\pi^{+}+\pi^{-})/2$	196±9	1.091±0.001	−0.16±0.03	170±9	35.77/22
		$(K^{+}+K^{-})/2$	207±10	1.031±0.002	0.97±0.01	28±3	9.02/22
		$(p+\bar{p})/2$	232±12	1.012±0.001	1.82±0.01	17±2	1.64/18
	62.4	$(\pi^{+}+\pi^{-})/2$	189±9	1.078±0.001	−0.25±0.02	208±9	103.95/6
		$(K^{+}+K^{-})/2$	212±10	1.031±0.001	0.99±0.01	35±3	1.50/6
		$(p+\bar{p})/2$	243±13	1.020±0.002	1.88±0.02	22±1	5.98/11
	130	$(\pi^{+}+\pi^{-})/2$	190±9	1.078±0.002	−0.26±0.01	245±9	122.72/6
		$(K^{+}+K^{-})/2$	213±10	1.031±0.003	1.00±0.01	44±3	2.23/8
		$(p+\bar{p})/2$	247±13	1.021±0.002	1.87±0.02	23±1	20.75/8
	200	$(\pi^{+}+\pi^{-})/2$	192±9	1.080±0.003	−0.26±0.01	286±9	85.21/7
		$(K^{+}+K^{-})/2$	218±11	1.034±0.002	1.11±0.02	49±3	0.42/6
		$(p+\bar{p})/2$	250±14	1.024±0.002	1.93±0.01	28±1	27.56/12
ALICE Pb-Pb	2760	$(\pi^{+}+\pi^{-})/2$	230±10	1.140±0.001	−0.16±0.00	709±11	155.11/37
		$(K^{+}+K^{-})/2$	251±13	1.067±0.002	1.09±0.02	109±6	4.63/32
		$(p+\bar{p})/2$	300±14	1.043±0.001	1.86±0.03	32±3	22.39/38
	5020	$\pi^{+}+\pi^{-}$	231±11	1.138±0.001	−0.15±0.01	1899±30	153.36/36
			999±18				
		$K^{+}+K^{-}$	250±13	1.067±0.001	1.21±0.01	269±10	5.95/32
			1100±20				
		$p+\bar{p}$	321±14	1.045±0.001	1.77±0.02	72±4	19.51/27
			999±16				
ALICE Xe-Xe	5440	$\pi^{+}+\pi^{-}$	238±12	1.140±0.002	−0.15±0.01	1057±33	21.89/36
		$K^{+}+K^{-}$	260±13	1.068±0.002	1.08±0.02	168±11	1.49/27
		$p+\bar{p}$	327±14	1.040±0.001	1.71±0.04	49±3	11.75/30

**Table 2:** Values of $T_{1}$, $T_{2}$, *q*, $a_{0}$, $N_{0}$, $\chi^{2}$, and ndof corresponding to the dashed curves in Figure 1, Figure 2 and Figure 3.

Collab.	$\sqrt{s_{\mathrm{NN}}}$ (GeV)	Particle	$T_{1}$, $T_{2}$ (MeV)	*q*	$a_{0}$	$N_{0}$	$\chi^{2}$/ndof
E866/E895 Au-Au	2.7	$\pi^{+}$	139±4	1.069±0.003	−0.49±0.01	12±2	12.31/19
		$K^{+}$	145±7	1.010±0.004	0.46±0.01	0.056±0.001	3.77/6
		*p*	183±4	1.005±0.001	1.55±0.01	76±6	148.48/36
	3.32	$\pi^{+}$	159±4	1.078±0.003	−0.45±0.01	28±2	62.79/24
		$K^{+}$	150±6	1.013±0.003	0.47±0.02	2.19±0.01	2.14/8
		*p*	194±5	1.005±0.002	1.66±0.01	69±3	244.84/36
	3.84	$\pi^{+}$	159±4	1.077±0.003	−0.42±0.02	37±6	45.43/19
		$K^{+}$	168±8	1.023±0.005	0.69±0.02	4.59±0.01	0.94/7
		*p*	195±5	1.005±0.002	1.64±0.02	61±5	310.66/36
	4.3	$\pi^{+}$	162±6	1.080±0.003	−0.42±0.02	46±9	56.47/16
		$K^{+}$	173±10	1.026±0.002	0.72±0.02	7.20±0.02	0.81/5
		*p*	202±7	1.007±0.003	1.74±0.02	59±9	74.66/36
E802 Au-Au	5.03	$\pi^{+}$	183±4	1.092±0.001	−0.23±0.01	53±6	164.90/30
		$K^{+}$	175±6	1.026±0.001	0.72±0.01	12±3	4.58/7
		*p*	205±7	1.007±0.003	1.74±0.03	62±5	65.21/25
NA49 Pb-Pb	6.3	$\pi^{+}$	185±4	1.093±0.001	−0.42±0.02	72±6	328.26/12
		$K^{+}$	175±6	1.026±0.002	0.78±0.01	100±2	30.95/6
		*p*	205±7	1.007±0.003	1.73±0.03	20±1	6.79/10
STAR Au-Au	7.7	$(\pi^{+}+\pi^{-})/2$	185±7	1.079±0.001	−0.25±0.01	91±2	54.50/22
		$(K^{+}+K^{-})/2$	190±9	1.026±0.005	1.03±0.01	14±3	1.90/16
		$(p+\bar{p})/2$	216±10	1.007±0.002	1.82±0.01	27±1	1.33/11
	11.5	$(\pi^{+}+\pi^{-})/2$	187±7	1.083±0.001	−0.21±0.01	120±5	41.38/22
		$(K^{+}+K^{-})/2$	194±9	1.029±0.003	0.99±0.01	19±3	1.03/19
		$(p+\bar{p})/2$	216±11	1.007±0.001	1.82±0.01	23±1	1.36/19
	14.5	$(\pi^{+}+\pi^{-})/2$	190±7	1.084±0.001	−0.20±0.02	141±9	3.74/24
		$(K^{+}+K^{-})/2$	191±9	1.025±0.006	0.97±0.01	22±3	0.81/14
		$(p+\bar{p})/2$	220±12	1.010±0.001	1.82±0.01	21±1	0.30/21
	19.6	$(\pi^{+}+\pi^{-})/2$	192±8	1.089±0.001	−0.18±0.03	150±6	39.67/21
		$(K^{+}+K^{-})/2$	201±9	1.026±0.003	0.96±0.01	24±4	17.06/22
		$(p+\bar{p})/2$	222±11	1.011±0.001	1.81±0.02	19±1	2.27/18
	27	$(\pi^{+}+\pi^{-})/2$	193±8	1.091±0.001	−0.18±0.01	164±6	27.36/21
		$(K^{+}+K^{-})/2$	203±9	1.028±0.003	0.99±0.01	26±3	10.01/20
		$(p+\bar{p})/2$	225±11	1.011±0.002	1.82±0.02	19±1	4.67/18
	39	$(\pi^{+}+\pi^{-})/2$	198±9	1.091±0.001	−0.16±0.03	176±9	59.05/22
		$(K^{+}+K^{-})/2$	208±10	1.031±0.002	0.97±0.01	28±3	9.05/22
		$(p+\bar{p})/2$	232±12	1.012±0.001	1.82±0.01	17±2	1.65/18
	62.4	$(\pi^{+}+\pi^{-})/2$	189±9	1.078±0.001	−0.25±0.02	208±9	97.82/6
		$(K^{+}+K^{-})/2$	212±10	1.031±0.001	1.00±0.01	35±3	1.50/6
		$(p+\bar{p})/2$	243±13	1.020±0.002	1.88±0.02	21±1	16.62/11
	130	$(\pi^{+}+\pi^{-})/2$	190±9	1.078±0.002	−0.26±0.01	248±9	143.34/6
		$(K^{+}+K^{-})/2$	213±10	1.031±0.003	1.00±0.01	44±3	2.25/8
		$(p+\bar{p})/2$	247±13	1.021±0.002	1.87±0.02	23±1	19.97/8
	200	$(\pi^{+}+\pi^{-})/2$	192±9	1.080±0.003	−0.26±0.01	288±9	111.25/7
		$(K^{+}+K^{-})/2$	218±11	1.034±0.002	1.12±0.02	48±3	0.42/6
		$(p+\bar{p})/2$	250±14	1.024±0.002	1.93±0.01	28±1	28.32/12
ALICE Pb-Pb	2760	$(\pi^{+}+\pi^{-})/2$	230±10	1.140±0.001	−0.16±0.01	709±11	155.11/37
		$(K^{+}+K^{-})/2$	251±13	1.067±0.002	1.09±0.02	109±6	4.64/32
		$(p+\bar{p})/2$	300±14	1.043±0.001	1.86±0.03	32±3	22.50/38
	5020	$\pi^{+}+\pi^{-}$	231±11	1.138±0.001	−0.15±0.01	1899±30	153.36/36
			999±18				
		$K^{+}+K^{-}$	250±13	1.067±0.001	1.21±0.01	269±10	5.94/32
			1100±20				
		$p+\bar{p}$	321±14	1.045±0.001	1.77±0.02	72±4	19.49/27
			999±16				
ALICE Xe-Xe	5440	$\pi^{+}+\pi^{-}$	238±12	1.140±0.002	−0.15±0.01	1057±33	21.89/36
		$K^{+}+K^{-}$	260±13	1.068±0.002	1.08±0.02	168±11	1.49/27
		$p+\bar{p}$	327±14	1.040±0.001	1.71±0.04	49±3	11.74/30

**Table 3:** Values of intercepts, slopes, and $\chi^{2}$ for the solid lines in Figure 5 and Figure 6, where ndof = 1, which is not shown in the table. The units of the intercepts in Figure 5 and Figure 6 are GeV and GeV/*c*, respectively. The units of the slopes in Figure 5 and Figure 6 are $c^{2}$ and *c*, respectively.

Figure	Relation	System	$\sqrt{s_{\mathrm{NN}}}$ (GeV)	Intercept	Slope	$\chi^{2}$
Figure 5a	$T-m_{0}$	Au-Au	2.7	0.117±0.002	0.067±0.002	1.08
			3.32	0.132±0.001	0.060±0.003	4.50
			3.84	0.143±0.002	0.053±0.003	0.43
Figure 5b	$T-m_{0}$	Au-Au	4.3	0.145±0.002	0.059±0.004	0.14
			5.03	0.164±0.002	0.040±0.003	2.14
		Pb-Pb	6.3	0.168±0.001	0.034±0.004	0.24
Figure 5c	$T-m_{0}$	Au-Au	7.7	0.171±0.002	0.046±0.003	0.48
			11.5	0.176±0.002	0.041±0.003	0.36
			14.5	0.176±0.001	0.044±0.004	1.32
Figure 5d	$T-m_{0}$	Au-Au	19.6	0.182±0.003	0.042±0.004	0.11
			27	0.184±0.003	0.043±0.004	0.13
			39	0.188±0.003	0.046±0.004	0.18
Figure 5e	$T-m_{0}$	Au-Au	62.4	0.179±0.003	0.068±0.001	0.01
			130	0.179±0.003	0.072±0.004	0.03
		Au-Au	200	0.182±0.004	0.073±0.004	0.01
Figure 5f	$T-m_{0}$	Pb-Pb	2760	0.214±0.003	0.089±0.004	0.45
			5020	0.208±0.003	0.114±0.003	1.84
		Xe-Xe	5440	0.216±0.003	0.113±0.003	1.23
Figure 6a	$\langle p_{T}\rangle -\bar{m}$	Au-Au	2.7	0.117±0.004	0.347±0.004	0.93
			3.32	0.106±0.004	0.379±0.005	2.52
			3.84	0.136±0.005	0.363±0.005	0.22
Figure 6b	$\langle p_{T}\rangle -\bar{m}$	Au-Au	4.3	0.125±0.004	0.387±0.005	0.17
			5.03	0.135±0.004	0.390±0.005	0.94
		Pb-Pb	6.3	0.155±0.005	0.369±0.004	0.06
Figure 6c	$\langle p_{T}\rangle -\bar{m}$	Au-Au	7.7	0.145±0.005	0.403±0.005	0.01
			11.5	0.156±0.005	0.395±0.007	0.01
			14.5	0.144±0.005	0.407±0.006	0.16
Figure 6d	$\langle p_{T}\rangle -\bar{m}$	Au-Au	19.6	0.150±0.004	0.408±0.005	0.01
			27	0.152±0.004	0.411±0.006	0.01
			39	0.148±0.004	0.423±0.006	0.21
Figure 6e	$\langle p_{T}\rangle -\bar{m}$	Au-Au	62.4	0.106±0.003	0.467±0.006	0.04
			130	0.102±0.003	0.472±0.008	0.04
			200	0.098±0.003	0.484±0.008	0.01
Figure 6f	$\langle p_{T}\rangle -\bar{m}$	Pb-Pb	2760	0.089±0.002	0.528±0.006	0.01
			5020	0.082±0.002	0.539±0.008	0.01
		Xe-Xe	5440	0.091±0.002	0.532±0.009	0.01

**Table 4:** Values of intercepts, slopes, and $\chi^{2}$ for the dashed lines in Figure 5 and Figure 6.

Figure	Relation	System	$\sqrt{s_{\mathrm{NN}}}$ (GeV)	Intercept	Slope	$\chi^{2}$
Figure 5a	$T-m_{0}$	Au-Au	2.7	0.126±0.002	0.056±0.002	1.79
			3.32	0.144±0.001	0.046±0.003	5.91
			3.84	0.150±0.002	0.046±0.003	0.48
Figure 5b	$T-m_{0}$	Au-Au	4.3	0.152±0.002	0.051±0.004	0.45
			5.03	0.172±0.002	0.029±0.003	3.10
		Pb-Pb	6.3	0.178±0.001	0.024±0.004	0.98
Figure 5c	$T-m_{0}$	Au-Au	7.7	0.176±0.002	0.040±0.003	0.75
			11.5	0.180±0.002	0.037±0.003	0.32
			14.5	0.180±0.001	0.039±0.004	1.37
Figure 5d	$T-m_{0}$	Au-Au	19.6	0.185±0.003	0.038±0.004	0.15
			27	0.186±0.003	0.040±0.004	0.14
			39	0.190±0.003	0.043±0.004	0.19
Figure 5e	$T-m_{0}$	Au-Au	62.4	0.179±0.003	0.068±0.001	0.01
			130	0.179±0.003	0.072±0.004	0.03
		Au-Au	200	0.182±0.004	0.073±0.004	0.01
Figure 5f	$T-m_{0}$	Pb-Pb	2760	0.214±0.003	0.089±0.004	0.45
			5020	0.208±0.003	0.114±0.003	1.84
		Xe-Xe	5440	0.216±0.003	0.113±0.003	1.23
Figure 6a	$\langle p_{T}\rangle -\bar{m}$	Au-Au	2.7	0.114±0.004	0.349±0.004	0.99
			3.32	0.109±0.004	0.376±0.005	2.31
			3.84	0.102±0.005	0.387±0.005	0.01
Figure 6b	$\langle p_{T}\rangle -\bar{m}$	Au-Au	4.3	0.120±0.004	0.389±0.005	0.27
			5.03	0.142±0.004	0.379±0.005	1.06
		Pb-Pb	6.3	0.151±0.005	0.372±0.004	0.10
Figure 6c	$\langle p_{T}\rangle -\bar{m}$	Au-Au	7.7	0.143±0.005	0.403±0.005	0.01
			11.5	0.152±0.005	0.398±0.007	0.01
			14.5	0.143±0.005	0.408±0.006	0.15
Figure 6d	$\langle p_{T}\rangle -\bar{m}$	Au-Au	19.6	0.152±0.004	0.407±0.005	0.01
			27	0.151±0.004	0.412±0.006	0.01
			39	0.148±0.004	0.422±0.006	0.79
Figure 6e	$\langle p_{T}\rangle -\bar{m}$	Au-Au	62.4	0.106±0.003	0.466±0.006	0.03
			130	0.101±0.003	0.472±0.008	0.04
			200	0.098±0.003	0.484±0.008	0.01
Figure 6f	$\langle p_{T}\rangle -\bar{m}$	Pb-Pb	2760	0.090±0.002	0.529±0.006	0.01
			5020	0.083±0.002	0.539±0.008	0.01
		Xe-Xe	5440	0.090±0.002	0.532±0.009	0.01

## Data Availability

The data used to support the findings of this study are included within the article and are cited at relevant places within the text as references.
